# PROTOCOL: Effectiveness of interventions for improving social inclusion outcomes for people with disabilities in low‐ and middle‐income countries: A systematic review

**DOI:** 10.1002/cl2.1191

**Published:** 2021-07-29

**Authors:** Ashrita Saran, Xanthe Hunt, Howard White, Hannah Kuper

**Affiliations:** ^1^ Campbell Collaboration Delhi India; ^2^ Stellenbosch University Cape Town South Africa; ^3^ Campbell Collaboration New Delhi India; ^4^ International Centre for Evidence on Disability London School of Hygiene & Tropical Medicine London UK

## Abstract

The objectives of this review are to: (1) examine the effectiveness of interventions for improving social inclusion outcomes for people with disabilities (physical, visual, hearing, intellectual or mental health conditions) in low‐ and middle‐income countries (LMICs); and (2) to critically appraise the confidence in study finding of the included studies. Key questions include: (1) Are interventions to improve social inclusion outcomes for people with disabilities in LMICs effective, and what is the quality of evidence base? (2) What types of intervention, or intervention design features, are most effective in improving social inclusion outcomes for people with disabilities in LMICs? (3) Which interventions appear most effective for different categories of disability? (4) What are the barriers to people with disabilities participating in interventions to improve their social inclusion outcomes? And what factors facilitate participation in, and the success of, such interventions?

## 1 BACKGROUND

### 1.1 The problem, condition or issue

Social inclusion is multifaceted and most commonly refers to inclusion in social, political, cultural and economic dimensions of life (Khan et al., 2015). A UN report on the World Social Situation defines social inclusion as the “process of improving the terms of participation in society, particularly for people who are disadvantaged, through enhancing opportunities, access to resources, voice and respect for rights” (UN, 2016).

On a global level, 80% of people with disabilities live in low‐ and middle‐income countries (LMICs) WHO, World report on disability, 2011. People with disabilities are greatly over‐represented among the most marginalised in society and often experience stigmatising attitudes, norms and behaviours. This stigma, coupled with inaccessible environments and systems and institutional barriers (e.g., lack of antidiscrimination legislation), may result in discrimination of people with disabilities, and potentially their families, so that they are not able to enjoy their rights on an equal basis with others. Consequently, people with disabilities on average have lower educational attainment, poorer health, lower economic opportunities and are at increased risk of poverty.

Social exclusion impacts individuals in diverse ways depending on their impairment type, gender, socioeconomic and cultural background, and other characteristic and contexts. For example, older people with disabilities often experience discrimination based upon both their disability and age, and older women may be even further disadvantaged. People with certain impairment types may face particularly high levels of discrimination. For instance, in many parts of the world people with albinism are often targeted as a result of deep‐rooted discriminatory beliefs, such as that their body parts can bring good fortune. Societal stigma can result in people with psychosocial and intellectual disabilities being segregated, constrained in their homes, or institutionalised.

Participation of people with disabilities in education, economic and politics is low when compared to people without disabilities (WHO, 2010). Health access and outcomes are also worse, on average, and many are poor as compared to people without disabilities (Banks et al., 2017; Bright & Kuper, 2018). These difficulties are both cause and consequence of social exclusion and arise as people with disabilities experience barriers in accessing services that others have long taken for granted, including health, education, employment and transport (Jones et al., 2012). These difficulties are exacerbated in less advantaged communities and increase the risk of social exclusion and poverty (WHO, 2010). These exclusions are contrary to the UN Convention on the Rights of Persons with Disabilities (UNCRPD), which supports the fulfilment of rights for persons with disabilities, across diverse areas, including education, employment and social participation.

Education supports skill development and schools are a crucial setting for developing social networks, peer relationships, friendships and influential linkages that may further lead to job opportunities or promote entrepreneurship (Hanushek & Wößmann, 2007). Similarly, employment facilitates friendship and engagement in society and helps promotes human dignity and social cohesion. Fulfilling the rights to education of children with disabilities and right to livelihood inclusion may also help other rights to be met—for instance, the schools and the workplace are a key provider of healthcare such as school‐based dissemination of food or drugs and receipt of social protection may help health care costs to be met.

Social inclusion of people with disabilities is recognised as a fundamental right in the UNCRPD, including in “participation in cultural life, recreation, leisure, and sport” (article 30) and in article 29 on participation in political and public life. Furthermore, without social inclusion other rights (e.g., right to education) may not be realised. The Sustainability Development Goals (SDGs) are also relevant to this issue (UN, 2015). SDG4 “Guaranteeing equal and accessible education by building inclusive learning environments and providing the needed assistance for persons with disabilities”, SDG 8 is to “Promote sustained, inclusive and sustainable economic growth, full and productive employment and decent work for all”, SDG 10 “Emphasizing the social, economic and political inclusion of persons with disabilities” and SDG 11 “Creating accessible cities and water resources, affordable, accessible and sustainable transport systems, providing universal access to safe, inclusive, accessible and green public spaces”. As long as people with disabilities are excluded from equal participation in all aspects of life, the SDGs arguably cannot be achieved.

Wider society also benefits from the valuable contributions that people with disabilities make. Further, meaningful inclusion of people with disabilities, for example, in arts, sports, and community processes, can challenge stigmatising attitudes and norms and, in turn, reduce discrimination and social exclusion. On an individual level, social inclusion of people with disabilities is important for many personal development reasons, including promoting health, well‐being, self‐esteem, and dignity, and strengthening social connections and economic opportunities.

Despite the benefits of social inclusion, there is evidence from LMICs that people with disabilities face widespread social exclusion, stigma, and discrimination. There is evidence from LMICs that people with disabilities face widespread social exclusion, stigma, and discrimination and violence (Jones et al., 2012). For instance, studies conducted in India, Cameroon, and Guatemala show that adults with disabilities face greater participation restrictions in interpersonal relationships and social, community, and civic with disabilities and lack of opportunity for engagement in activities outside of the home (Sheppard et al., 2018). Research from humanitarian contexts conducted in refugee camps in Tanzania and conflict‐affected areas of Ukraine shows high levels of social isolation among older people all aspects of life—political, economic, social, cultural, and civil or any other field—and includes denial of reasonable accommodation.

Barriers that limit social inclusion of people with disabilities include physical barriers such as inaccessible transport and buildings (e.g., community centres, sport facilities) and information barriers (e.g., lack of sign‐language interpreters at cultural events). Another core reasons for social exclusion is stigma and discrimination. Discrimination on the basis of disability means any distinction, exclusion or restriction that has the purpose or effect of preventing people with disabilities access to their rights (MacKay, 2006), and is widespread (Mactaggart et al., 2016). People with disabilities also experience stigmatising attitudes, which act as further barriers to inclusion. These are inaccurate perceptions and beliefs that can be widespread in society and can often result in and underpin exclusion, and sometimes exploitation, abuse and violence (WHO, 2010) (Jones et al., 2012). People who are stigmatised are made to feel ashamed, and stigma is often one of the driving factors behind discrimination against people with disabilities and consequent social exclusion (Bond Disability and Development Group, 2017). For example, prejudice and misconceptions result in people with disabilities being discriminated against by being denied opportunities—including opportunities to establish relationships, express their sexuality, marry, and have families (WHO/UNFPA, 2009). The families and carers of people with disabilities are also often stigmatised or discriminated against by association (DFID, 2018).

### 1.2 The intervention

This is a problem‐oriented review, and so is not restricted to a single intervention. Rather all interventions which may improve social inclusion of people with disabilities are included. We consider the scope of social inclusion in line with the WHO's Community Based Rehabilitation (CBR) Guidelines (WHO, 2010). CBR, which is promoted by the WHO to improve the lives of people with disabilities, has “Social” as one of its five pillars (WHO, 2010). Within the “social” pillar of the CBR matrix, there are five specific components which we use to classify interventions: personal assistance, relationship, marriage and family, culture and arts, recreation, leisure and sports and justice. Each of these intervention categories has specific interventions which are named in Table 1 (e.g., formal personal assistance and support, informal personal assistance and support). Therefore, the CBR will serve as a guiding framework for the intervention categories, as listed below, to realize the full inclusion and empowerment of persons with disabilities. We have added two additional categories to the CBR framework social pillar, namely Assistive Technologies (AT) and Rehabilitation, and Policies. We will consider interventions that specifically target people with disabilities, as well as mainstream programmes that are inclusive of people with disabilities.

**Table 1:** Intervention and subintervention categories

Intervention category	Intervention subcategory	Description
Personal assistance	Formal personal assistance and support (including training)	Formal assistance may be provided on a formal basis by governmental and nongovernmental organisations and the private sector. Allowances, such as disability pensions, guardianship awards or caregiver allowances, may be available to fund personal assistance (Khasnabis et al., 2010)
	Informal personal assistance and support (including training)	Informal assistance includes assistance by family members, friends, neighbours and/or volunteers (Khasnabis et al., 2010)
Relationship, marriage and family	Networking and social support	Includes linking people with disabilities to appropriate support networks in the community, for example, disabled people's organizations and self‐help groups (Khasnabis et al., 2010)
	Improving community attitude	It involve working with the media to promote positive images and role models of people with disabilities; and information on services available (Khasnabis et al., 2010)
	Community living	It involves interventions to support people with disabilities to access their preferred living arrangements and support people with disabilities who are homeless to find appropriate accommodation, preferably in the community
	Social and communication skill training	Social skills training is a therapeutic approach used to improve interpersonal relations. The therapy focuses on verbal and nonverbal behaviours common in social relationships.
	Violence prevention interventions	This includes all the interventions to prevent violence such as raising awareness, establishing links to local stakeholders for support, access to health care services, and so forth (Khasnabis et al., 2010)
Culture and arts	Access and participation in cultural programs, arts, drama and theatres	People with disabilities enjoy access and participation to cultural materials in accessible formats; to television programmes, films, theatre and other cultural activities, in accessible formats; to places for cultural performances or services, such as theatres, museums, cinemas, libraries and tourism services, and, as far as possible, to monuments and sites of national cultural importance (UNCRPD, 2006)
	Access and participation in religious activities	People with disabilities enjoy access and participation in religious and spiritual activities in accessible formats, for example, making prayers, songs, chanting, and sermons accessible with signed translation, and making religious texts available in large print, audio and Braille; Places of worship are physically accessible and that religious practices are modified to accommodate people with disabilities (UNCRPD, 2006)
Recreation, leisure and sports	Access and participation in sports events	This includes strategies that encourages people with disabilities to have access and provide opportunities to participate in mainstream sporting activities at all levels through inclusive sports event; have an opportunity to organise, develop and participate in disability‐specific sporting and recreational activities through provision of support and links with DPOs for people with disabilities, assisting them to develop strategic, national and international partnerships and have access to adapted sports equipment (Khasnabis et al., 2010)
	Access and participation in recreation and leisure	This includes strategies that encourages people with disabilities to have access and provide opportunities to participate in mainstream sporting activities to provide opportunities to participate actively or passively in recreation, tourism and leisure (Khasnabis et al., 2010)
Justice	Accessibility of egal system and justice	Accessibility”, in this publication refers to a feature or quality of any physical or virtual environment, space, facility or service that is capable of accommodating the needs of people with disabilities to understand, get access to or interact with legal system. Accessibility also refers to technical standards that are mandated nationally or internationally for the design and construction of a physical or virtual environment, space, facility and service. Examples include accessible built infrastructure of courts such as ramps, and so forth
	Access to legal system and justice	Refers to people's ability to access the systems, procedures, information, and locations used in the administration of justice (Lord & Stein, 2008) This includes activities such as legal awareness through DPOs and media, legal aid
Assistive technology and rehabilitation	Assistive technology	This involves all the activities for the detection, assessment and treatment to stop the progression of a health condition in people with disabilities
	Rehabilitation	Rehabilitation is a process intended to eliminate or at least minimise—restrictions on the activities of people with disabilities, permitting them to become more independent and enjoy the highest possible quality of life (Bailey & Angell, 2005). This will include activities as provision of mobility, hearing, visual devices, and therapies to use these devices
	Medical care	Provision of medical services to ensure that people with disabilities can access services designed to identify, prevent, minimize and/or correct health conditions and impairments (Khasnabis et al., 2010)
Policies and programmes	International legislations and policies	These include international legislations and policies through which countries abolish discrimination against persons with disabilities and eliminate barriers towards the full enjoyment of their rights and their inclusion in society (UN Department of Economic and Social Affairs)
	Social inclusion policies	This includes inclusive policies on employment, educational and provision of housing and accommodation to people with disabilities

The five components in social pillar of CBR are:

**Personal assistance:** Personal assistance may be helpful because of environmental factors (e.g., when the environment is inaccessible), and when people with disabilities have impairments and functional difficulties that make it difficult to carry out activities and tasks on their own. Personal assistance interventions include formal personal assistance and support, informal personal assistance and support, and personal assistance training (UNCRPD, 2007).

**Relationship, marriage and family:** This component highlights the importance of supporting people with disabilities to establish relationships, marry and become parents if they choose. It includes interventions such as peer support, social networks, appropriate living conditions, community facilities and violence prevention interventions (UNCRPD, 2006).

**Culture and arts:** This component enables people with disabilities to enjoy access to cultural materials in accessible formats; television programmes, films, theatre and other cultural activities, in accessible formats; places for cultural performances or services, such as theatres, museums, cinemas, libraries and tourism services. The interventions range from inclusive art education, sign‐language interpreters, cultural programs, theatres, arts and dramas, complementary therapy in the form of art and music and participation in religious activities (UNCRPD, 2006).

**Recreation, leisure and sports:** This component supports people with disabilities to participate both actively and as spectators in recreational, leisure and sporting activities on an equal basis with others. Interventions include networking and capacity building, organisation of inclusive sports events, provision of adapted sports equipment, recreation and sports clubs, community concerts and media, sports based disability programs (UNCRPD, 2006).

**Justice:** This component enables people with disabilities to have access to justice on an equal basis with others to ensure full enjoyment and respect of human rights. Interventions include inheritance rights, provision of procedural and age‐appropriate accommodations, included as witnesses, in all legal proceedings, included at investigative and other preliminary stages (UNCRPD, 2006).

### 1.3 How the intervention might work

It is important to consider the barriers to social inclusion experienced by people with disabilities, to identify how these may be overcome. People with disabilities are not a homogenous group, and the reasons for exclusion will vary for women and men, in different settings, and for people with different impairment types. Nevertheless, barriers can be broadly categorised as being experienced at the level of the individual, the community, the system.

**Individual‐level barriers** include lower level of social and communication skill training, lack of personal assistance and support; lack of access to adapted sports equipment, lack of braille or versions for people who use screen readers. Internalised barriers can affect dignity and confidence of people with disabilities, for instance, societal stigma can result in people with disabilities being segregated, constrained in their homes, or institutionalised. This can further lead to denied opportunities—including opportunities to establish relationships, express their sexuality, marry, and have families.

**Community‐level barriers** include physical barriers such as inaccessible transport and buildings (e.g., community centres, sport facilities) and information barriers (e.g., lack of sign‐language interpreters at cultural events), negative thoughts beliefs and attitudes by community towards participation of people with disabilities in sports and leisure activities, lack of advocacy and volunteer groups (DPOs), lack of information on inclusive activities and events.

**System‐level barriers** include inadequate resource allocation to support personal assistance of people with disabilities, discriminatory legislation and policies that exacerbate the exclusion of people with disabilities from decision‐making processes and other areas of life injustice preventing full participation of people with disabilities. Lack of awareness or enforcement of existing laws and regulations that require programs and activities be accessible to people with disabilities.

**Figure 1:**
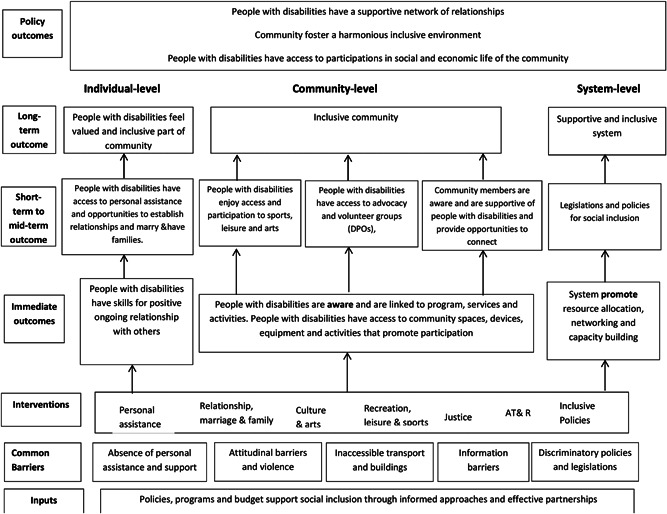
Logic mode concerning the effectiveness of interventions for improving social inclusion for peope with disabilities

Approaches to improve social inclusion and outcomes for people with disabilities must act by targeting the barriers that they experience. In other words, they must operate at the level of the individual (e.g., personal assistance training and support), community (e.g., access to buildings such community centres and recreation centres) and system (e.g., improving policy and legislation) Figure 1. Programs or activities may aim to operate at different levels concurrently. aimed at more than one target group or for more than one, one level of barriers can be combined to capture the program's goal. For example, FANDIC (Friends of Children with Disability for their Integration into the Community) intended to provide individual opportunities to develop physical and artistic abilities (individual‐level), to integrate children with disabilities into the community (community‐level), also provide individual opportunities to develop physical and artistic abilities (community‐level). It may involve coordination to increase awareness about disability at various levels including the individual, community, organisational and governmental (system‐level).

**Individual‐level interventions** include activities such as provision of mobility and communication aids or assistive devices; build social skills, inclusive sports events, rehabilitation and treatment; people with disabilities are assisted to attend where necessary i.e. transport, cultural support, and so forth. Efforts to change attitudes are also important, so that people with disabilities are seen past the impairment; discrimination; fear; bullying to achieve equality of opportunities and societal integration.

**Community‐level interventions** include adaptations of buildings and transport to be accessible, services and programs that are accessible to people with disabilities and aim to raise awareness, reduce stigma, interventions to prevent violence such as raising awareness in community through media, establishing links to local stakeholders for support, access to health care services, mainstreaming education, sports, recreation and leisure, and building a welcoming and inclusive community.

**System‐level interventions** include effective legislation for social inclusion such as inheritance rights, budget allocation for personal assistance, inclusive events, media awareness on the rights of people with disabilities.

### 1.4 Why it is important to do this review

Social inclusion of people with disabilities is recognised as a fundamental right in the UNCRPD, including in “participation in cultural life, recreation, leisure, and sport” (article 30) and in article 29 on participation in political and public life. Furthermore, without social inclusion other rights (e.g., right to education) may not be realised. Social inclusion is also fundamental to implementing the 2030 Agenda; as long as people with disabilities are excluded from equal participation in all aspects of life, the SDGs arguably cannot be achieved. Wider society also benefits from the valuable contributions that people with disabilities make. Various approaches are used to promote social inclusion for people with disabilities, with the ambition that “People with disabilities have meaningful social roles and responsibilities in their families and communities, and are treated as equal members of society” (Khasnabis, Al Jubah, Brodtkorb, Chervin, P Goerdt).

Several relevant systematic reviews and protocols exist that are relevant to the topic, but none which would address the stated objectives of this review.

– Almerie et al. (2015) conducted a review of social skills programmes for people with schizophrenia and identified 13 RCTs. They concluded that social skills training may be effective at improving the social skills of people with schizophrenia, but that the data is limited and of very low quality.
– Mikton et al conducted a systematic review of the effectiveness of interventions to prevent and respond to violence against persons with disabilities Mikton et al. (2014). They identified 10 eligible studies, of which only one was from an LMIC. The studies were rated as poor quality, and the authors concluded “The current evidence base offers little guidance to policy makers, program commissioners, and persons with disabilities for selecting interventions”.
– Velema and colleagues assessed the evidence for effectiveness of rehabilitation‐in‐the‐community programmes, and concluded that CBR activities result in social processes that change the way community members view persons with disabilities, increase their level of acceptance and social inclusion and mobilise resources to meet their needs. However, the individual studies included in the review did not focus on improving social inclusion (Velema et al., 2008).
– REA on social inclusion Rapid Evidence Assessment of “What Works” to Improve Social Inclusion and Empowerment for People with Disabilities in Low and Middle Income Countries. International Centre for Evidence in Disability, London School of Hygiene and Tropical Medicine and Campbell Collaboration 2018.

Even in presence of the international efforts and despite the benefits of social inclusion, there is currently a lack of evidence from LMICs on the effectiveness of interventions on adopting a disability inclusive approach to development, and these outcomes remain complex and difficult to quantify (Walton, 2012). Hence, evidence on “what works” to improve social inclusion of people with disabilities is needed to inform policy, practice, and further research.

## 2 OBJECTIVES

The objectives of this review are to: (1) examine the effectiveness of interventions for improving social inclusion outcomes for people with disabilities (physical, visual, hearing, intellectual or mental health conditions) in LMICs; and (2) to critically appraise the confidence in study finding of the included studies.

Key questions include:

1. Are interventions to improve social inclusion outcomes for people with disabilities in LMICs effective, and what is the quality of evidence base?
2. What types of intervention, or intervention design features, are most effective in improving social inclusion outcomes for people with disabilities in LMICs?
3. Which interventions appear most effective for different categories of disability?
4. What are the barriers to people with disabilities participating in interventions to improve their social inclusion outcomes? And what factors facilitate participation in, and the success of, such interventions?

## 3 METHODS

### 3.1 Criteria for considering studies for this review

#### 3.1.1 Types of studies

Impact evaluations:

Eligible study designs are defined on the basis of being a type of impact evaluation. Descriptive studies of various designs and methodologies are not included because they, unlike impact evaluations, cannot speak to the question of effect. To answer the question posed by this review “What works to improve social inclusion for people with disabilities in LMICs?”, evidence of effect is required. Hence, qualitative studies, process evaluations and cross‐sectional studies will not be eligible for inclusion.

Eligible designs include those in which one of the following is true:

a) Participants are randomly assigned (using a process of random allocation, such as a random number generation),
b) A quasi‐random method of assignment has been used,
c) Participants are nonrandomly assigned but matched on pretests and/or relevant demographic characteristics (using observables or propensity scores) and/or according to a cut‐off on an ordinal or continuous variable (regression discontinuity design),
d) Participants are nonrandomly assigned, but statistical methods have been used to control for differences between groups (e.g., using multiple regression analysis or instrumental variables regression),
e) The design attempts to detect whether the intervention has had an effect significantly greater than any underlying trend over time, using observations at multiple time points before and after the intervention (interrupted time‐series design),
f) Participants receiving an intervention are compared with a similar group from the past who did not (i.e., a historically controlled study), or
g) Observations are made on a group of individuals before and after an intervention, but with no control group (single‐group before‐and‐after study).

#### 3.1.2 Types of participants

The target populations are populations are people with disabilities living in LMICs. Population subgroups of interest include: women, vulnerable children (particularly children in care), conflict (conflict and post‐conflict settings), migrants, ethnic minority groups and people with different impairment types; visual impairment, hearing impairment, physical impairment and intellectual impairment. For studies with multiple population, we will include studies if one of the population subgroup is people with disabilities.

#### 3.1.3 Types of interventions

The goal of WHO's CBR social component is that people with disabilities have meaningful social roles and responsibilities in their families and communities and are treated as equal member of the society. There are no restrictions on comparators/comparison groups; however a study must have both an eligible intervention and an eligible outcome to be included. It focusses on improving social inclusion, which can be achieved through the intervention categories listed below:

#### 3.1.4 Types of outcome measures

Eligible outcomes will relate to the social inclusion pillar of the CBR matrix All outcomes will be relevant regardless of whether they are primary outcomes, or secondary outcomes. It is important to note that if the primary study does not have both an eligible intervention and an eligible outcome then it will be excluded. The outcomes are listed in Table 2 below.

3.2

**Duration of follow‐up**

Any duration of follow‐up will be included.

**Types of settings**

All settings will be eligible, provided that the study is situated within a LMIC, as defined by the World Bank (2018; https://datahelpdesk.worldbank.org/knowledgebase/articles/906519-world-bank-country-and-lending-groups).

### 3.3 Search methods for identification of studies

The search will comprise: (1) an electronic search of databases and sector‐specific websites, and (2) screening of all included studies in the instances where reviews are identified.

#### 3.3.1 Electronic searches

A search of the following electronic databases will be conducted by the authors.

- MEDLINE(R)
- Embase Classic+Embase
- PsycINFO
- CAB Global Health
- CINAHL
- ERIC
- Scopus
- Web of Science (Social Sciences Citation Index)
- WHO Global Health Index
- MEDLINE
- Embase
- PsychINFO
- CAB Global Health
- OVID
- ERIC
- CINAHL
- Ebsco
- PubMED

Search strategies will be tailored for each of the databases. The main search terms will be as follows.

**Population:** (disable* or disabilit* or handicapped) OR (physical* or intellectual* or learning or psychiatric* or sensory or motor or neuromotor or cognitive or mental* or developmental or communication or learning) OR (cognitive* or learning or mobility or sensory or visual* or vision or sight or hearing or physical* or mental* or intellectual*) adj2 (impair* or disabilit* or disabl* or handicap*) OR (communication or language or speech or learning) adj5 (disorder*) OR (depression or depressive or anxiety or psychiat* or well‐being or quality of life or self‐esteem or self perception) adj2 (impair* or disabilit* or disabl* or handicap*) OR mental health OR (schizophreni* or psychos* or psychotic or schizoaffective or schizophreniform or dementia* or alzheimer*) adj2 (impair* or disabilit* or disabl* or handicap*) OR (mental* or emotional* or psychiatric or neurologic*) adj2 (disorder* or ill or illness*) OR (autis* or dyslexi* or Down* syndrome or mongolism or trisomy 21) OR (intellectual* or educational* or mental* or psychological* or developmental) adj5 (impair* or retard* or deficien* or disable* or disabili* or handicap* or ill*) OR (hearing or acoustic or ear*) adj5 (loss* or impair* or deficien* or disable* or disabili* or handicap* or deaf*) OR (visual* or vision or eye* or ocular) adj5 (loss* or impair* or deficien* or disable* or disabili* or handicap* or blind*) OR (cerebral pals* or spina bifida or muscular dystroph* or arthriti* or osteogenesis imperfecta or musculoskeletal abnormalit* or musculo‐skeletal abnormalit* or muscular abnormalit* or skeletal abnormalit* or limb abnormalit* or brain injur* or amput* or clubfoot or polio* or paraplegi* or paralys* or paralyz* or hemiplegi* or stroke* or cerebrovascular accident*) adj2 (impair* or disabilit* or disabl* or handicap*) OR (physical* adj5 (impair* or deficien* or disable* or disabili* or handicap*) OR people with disabilities/or children with disabilities/or people with mental disabilities/or people with physical disabilities/OR abnormalities/or exp congenital abnormalities/or exp deformities/or exp disabilities/or exp malformations/OR exp mental disorders/or exp mental health/or learning disabilities/or paralysis/or paraparesis/or paraplegia/or poliomyelitis/or hearing impairment/or deafness/or people with hearing impairment/or vision disorders/or blindness/or people with visual impairment/

**Study design:** controlled clinical trial/or randomized controlled trial/or equivalence trial/or pragmatic clinical trial/or case‐control studies/or retrospective studies/or cohort studies/or follow‐up studies/or longitudinal studies/or prospective studies/or epidemiologic methods/or epidemiologic studies/or controlled before‐after studies/or cross‐sectional studies/or interrupted time series analysis/or control groups/or cross‐over studies/or double‐blind method/or matched‐pair analysis/or meta‐analysis as topic/or random allocation/or single‐blind method/or "retraction of publication"/or case reports/OR (random or placebo or single blind or double blind or triple blind or cohort or ((case or follow up or follow‐up) adj2 (control or series or report or study or studies)) or retrospective or (observ adj3 (study or studies)))

**Location:** Developing Countries OR Africa/or Asia/or Caribbean/or West Indies/or Middle East/or South America/or Latin America/or Central America/OR (Africa or Asia or Caribbean or West Indies or Middle East or South America or Latin America or Central America) OR ((developing or less* developed or under developed or underdeveloped or middle income or low* income or underserved or under served or deprived or poor*) adj (countr* or nation? or population? or world or state*)) OR ((developing or less* developed or under developed or underdeveloped or middle income or low* income) adj (economy or economies)) OR (low* adj (gdp or gnp or gross domestic or gross national)) OR (low adj3 middle adj3 countr*) OR (lmic or lmics or third world or lami countr*) OR transitional countr*

#### 3.3.2 Searching other resources

To ensure maximum coverage of unpublished literature, and reduce the potential for publication bias, we will search the following organisational websites and databases using the keyword search for unpublished grey literature.

- ILO
- DFID (including Research for Development [R4D])
- UNESCO
- WHO
- Disability Programme of the United Nations Economic and Social Commission for Asia and the Pacific (UNSCAP)
- United States Agency for International Development (USAID)
- Dissertation Abstracts, Conference Proceedings and Open Grey.
- Humanity and Inclusion (HI) http://www.hi-us.org/publications
- CBM https://www.cbm.org/Publications-252011.php
- Plan international https://plan-international.org/publications

### 3.4 Data collection and analysis

#### 3.4.1 Description of methods used in primary research

We will use EppiReviewer (https://eppi.ioe.ac.uk/) to help assess the search results. EPPI Reviewer is a web‐based software program for managing and analysing data for literature reviews and has been developed for all types of systematic review such as meta‐analysis, framework synthesis and thematic synthesis. In our review, EPPI Reviewer will be used for bibliographic management, screening, coding and data synthesis.

#### 3.4.2 Criteria for determination of independent findings

It is possible for studies to report multiple outcomes, or for researchers to publish several articles using data from the same sample. For proper statistical analyses, it is important to ensure that all studies come from independent samples. Therefore, all articles that meet the criteria for inclusion will be examined to identify situations where multiple articles analyzed data from the same sample. Multiple publications of the same study will be examined as a single study. If multiple methods are used to measure the same outcome within the same study, the reviewers will select the most relevant measure for analysis using the following decision rules:

• Outcomes measured via validated formal scales are more relevant than those measured using a single‐item question.
• Clinician‐rated outcome measures are more relevant than self‐reported measures.

As mentioned above, during extraction, special attention will be paid to ensure that multiple reports of the same study are not treated as multiple studies. Should a study contain multiple intervention arms, the reviewers will only extract data on the intervention and control groups that are eligible for this review. Should a multiarm study report multiple relevant intervention arms, the findings from the different arms will be reported and analysed separately.

#### 3.4.3 Selection of studies

Screening will be a two‐stage process of first screening by title and abstract and then full text. Screening will be undertaken independently by two screens, with a third‐party arbiter in case of disagreement. Unique references will be screened for relevance by title and abstract and full text by two independent reviewers. The screening checklist will also be reviewed by H. K. and H. W. Eligibility will be assessed using a predesigned form based on the inclusion criteria. Articles excluded at this stage will be reported in a Table 3 with reasons for exclusion. We will report interrater reliability for study identification. The screening process will be reported using a PRISMA flow chart. The screening will be done using the screening tool/checklist listed below.

#### 3.4.4 Data extraction and management

Two review authors (A. S. and X. H.) will independently code and extract data from included studies. A coding sheet will be piloted on several studies and revised as necessary. Disagreements will be resolved by consulting a third review author with extensive content and methods expertise (H. W. and H. K.), and will be reported. Data and information will be extracted on: available characteristics of participants, intervention characteristics and control conditions, research design, sample size, risk of bias and outcomes, and results. Extracted data will be stored electronically. Studies will be coded by intervention, outcomes and a range of filters such as study design and location. Summaries of the studies will be prepared by a separate team. The primary studies included in the systematic reviews will also be assessed for eligibility. As such, the systematic review does not include summarised findings of the systematic reviews to avoid duplication. This evidence assessment is based on studies reporting outcomes in the domain social inclusion. The list of studies coded as such will be screened for eligibility by Ashrita.

A summary of included studies will be prepared, in addition to the coding, which will include: (1) basic study characteristics, (2) narrative summary (including annotation of any negative effects), (3) summary of findings/results table, and (4) quality assessment. This coding will be conducted by pairs of coders, with comparison and discussion to resolve any discrepancies which arise. The studies will be grouped by outcomes, that is: social, skills for social inclusion, broad based social inclusion and participation measures and relationships. For each outcome a narrative summary will be prepared for the main themes and findings, including consideration of where there is strong evidence for effect, where there are evidence gaps, and the quality of the evidence.

Data will be extracted from the studies according to an extraction table, coding is added as an Annex 1.

#### 3.4.5 Assessment of risk of bias in included studies

This tool^1^ contains six criteria:

1. Study design (potential confounders taken into account): impact evaluations need either a well‐designed control group, preferably based on random assignment, or an estimation technique which controls for confounding and the associated possibility of selection bias.
2. Masking (RCTs only, also known as blinding): masking helps limit the biases which can occur if study participants, data collectors or data analysts are aware of the assignment condition of individual participants.
3. Presence of a power calculation: many studies may be underpowered, but it is difficult to assess without the inclusion in the study of a power calculation.
4. Attrition can be a major source of bias in studies, especially if these is differential attrition between the treatment and comparison group so that the two may no longer be balanced in preintervention characteristics. The US Institute of Education Sciences What Works Clearing House has developed standards for acceptable levels of attrition, in aggregate and the differential, which we will apply.^2^
5. Clear definition of disability: for a study to be useful the study population must be clear, which means that the type and severity of disability should be clearly defined, preferably with reference to a widely used international standard
6. Clear definition of outcome measures is needed to aid interpretation and reliability of findings and comparability with other studies. Studies should clearly state the outcomes being used with a definition and the basis on which they are measured, preferably with reference to a widely used international standard.
7. Baseline balance shows that the treatment and comparison groups are the same at baseline. Lack of balance can bias the results.

Confidence in study findings will be rated high, medium or low, for each of the criteria, applying the standards as shown in Table 4. Overall study quality will be the lowest rating achieved across the criteria—the weakest link in the chain principle.

**Table 2:** Outcome and outcome subcategories

Outcome category	Outcome subcategory	Description
Social	Social identity	Social identity is defined as that part of a person's self‐concept which derives from the knowledge of his or her membership in a social group (or groups) together with the value and emotional significance attached to that membership. Social identity can spur intergroup discrimination and other forms of intergroup conflict (Simon et al., 2008)
	Personal assistance	People with disabilities have individual support plans in place, have access to training to enable them to manage their personal assistance needs, or that support is available for families who provide personal assistance on an informal basis
Skills for social inclusion	Social and communication skills	Kratochwill and French (1984) view social skills as learned verbal and nonverbal behaviour performed within a specific social context of an aggressiveness‐shyness continuum, and view adjustment in relation to an individual's social perceptual accuracy (i.e., the ability to understand subtle nuances and define critical elements in social environment). Communication skills is the act of transferring information. It may be vocally (using voice), written (using printed or digital media such as books, magazines, websites or emails), visually (using logos, maps, charts or graphs) or nonverbally (using body language, gestures and the tone and pitch of voice). This includes availability and use of communication aids and speech and reading devices
	Social behaviour	Social behaviour can be defined as all behaviour that influences, or is influenced by, other members of the same species. The term thus covers all behaviour that tends to bring individuals together as well as all forms of aggressive behaviour (Grant, 1963). This includes conduct problems, peer problems, pro‐social behaviours
Broad based social inclusion and participation measure	Social inclusion	Social inclusion is defined as the process of improving the terms of participation in society, particularly for people who are disadvantaged, through enhancing opportunities, access to resources, voice and respect for rights (UN, 2010). These will include measures such as people with disabilities spending more time out of the house, and travelling further away from the house (as well as earning more and spending less time begging)
	Community integration	Community Integration is the opportunity to live in the community and be valued for one's uniqueness and abilities, like everyone else. (Salzer & Baron, 2014). Community integration is designed to help people with disabilities to optimise their personal, social and vocational competency to live successfully in the community
	Community participation	People with disabilities have access, accessibility and opportunities to participate in community activities such as leisure activities, such as hobbies, arts, and sports, political and civic activities or organizations and productive activities, like employment or education; consumption, or access to goods and services; religious and cultural activities and groups (McConkey & Abbott, 2006)
	Access to justice	People with disabilities get access to or interact with legal system
Relationships	Interpersonal and Family relationship	People with disabilities value relationships with family members, staff, friends, acquaintances, and intimate partners (Clarkson et al., 2009) and other people with disabilities, and feeling a sense of belonging to a network when they have different people fulfilling different needs (McVilly et al., 2006a). This also includes aspects of participation in household, behaviour of the family towards the people with disability (e.g., more sensitive to child's interests, responded more appropriately, expressed more warmth)
	Peer and community relationships	Community members are aware and accept that people with disabilities can have meaningful relationships, marry and have children (Community‐Based Rehabilitation: CBR Guidelines)
	Violence and abuse	People with disabilities are protected against violence, and all relevant stakeholders work together to address the issue (Community‐Based Rehabilitation: CBR Guidelines)

**Table 3:** Screening tool for effectiveness of interventions for improving social inclusion outcomes for people with disabilities

1.	Is the paper in English?	No	Exclude
		Yes	Continue to q2
2.	Is the paper about social inclusion interventions for people with disabilities living in low‐and‐middle income countries (LMICs)?	No	Exclude
		Yes	Continue to q3
3.	Does the study assess the impact of intervention on social inclusion* outcomes for people with disabilities (includes personal assistance, relationship, marriage and family, culture and arts, recreation, leisure and sports and justice)	No	Exclude
		Yes	Continue to q4
4.	Is the paper a quantitative evaluation reporting measures of eligible outcomes compared to the outcomes (1) in a comparison group (either with or without baseline outcome measures), (2) before versus after with no comparison group,	No	Exclude
		Yes	Include

*Note:* Social inclusion* process of improving the terms of participation in society for people with disabilities through enhancing opportunities, access to resources, voice and respect for rights.

**Table 4:** Study quality assessment criteria

Item		Criterion
1	Study design (potential confounders taken into account)	High confidence: RCT, RDD, ITT, instrumental variable
		Medium confidence: DiD with matching, PSM
		Low confidence: other matching
2	Blinding (RCTs only)	High confidence: any blinding or any mention of blinding
		Medium confidence: no blinding
		Low confidence is not used for this item
3	Losses to follow up are presented and acceptable*	High: Overall and differential attrition within WWC combined levels*
		Medium: Overall and differential attrition close to WWC combined levels*
		Low: Attrition not reported, OR falls well outside WWC acceptable combined levels*
4	Disability/impairment measure is clearly defined and reliable	High confidence: Clear definition, for example, Washington Group questions, detailed measure of impairment
		Medium confidence: Unclear definition OR Single question item only (e.g., are you disabled)
		Low confidence: No definition OR overall attrition > 50%
5	Outcome measures are clearly defined and reliable	High confidence: Clear definition using existing measure where possible
		Medium confidence: unclear definition
		Low confidence: no definition
6	Baseline balance (N.A. for before vs. after)	High confidence: RCT, RDD
		Medium confidence: Baseline balance test, imbalance on 5 or fewer measures
		Low confidence: No baseline balance test (except RCT) OR reported and significant differences on more than five measures. PSM without establishing common support.
	Overall confidence in study findings	High: RCT with high confidence on all items
		Medium: Medium or high confidence on all items
		Low: Low on any item

Where a study reports outcomes at more than one point in time it is possible that the study quality varies between those two points for two of the criteria: (1) an RCT may no longer be so if it used a waitlist or pipeline design so the control group has received the treatment (item 1), (2) there may be greater attrition rates at the later point in time. Hence in applying the tool an assessment is made for the earliest and latest outcome measures for items 1 and 4, and overall study quality assessed separately for the two points in time.

#### 3.4.6 Measures of treatment effect

We will collect effect sizes and conduct effect size calculations where published, but, as noted based on the findings of published REA on social inclusion, we do not expect that it will be possible to conduct a meta‐analysis, given the diversity of designs, methodologies, measures and rigour across studies in this area.

**Calculating effect sizes**

We will convert these effect sizes to a common metric and will present these in forest plots.

**Standardized mean difference statistic (d‐index**): For continuous outcomes, effects sizes with 95% confidence intervals (CIs) will be calculated, where means and standard deviations are available. If means and standard deviations are not available, we will calculate standardised mean differences (SMDs) from *F* ratios, *t* values, *χ*2 values and correlation coefficients, where available, using the methods suggested by Lipsey and Wilson (2001).

**Odds ratio family:** Studies reporting dichotomous data, in which mean outcomes are compared in the experimental and control (or comparison groups) will be summarised using the odds ratio derivative statistic. A 95% CI for the odds ratio, risk difference or risk ratio statistics will be used to report all effect sizes.

There are statistical approaches available to re‐express dichotomous and continuous data to be pooled together (Sánchez‐Meca et al., 2003). To calculate common metric odds ratios will be converted to SMD effect sizes using the Cox transformation. We will only transform dichotomous effect sizes to SMD if appropriate.

When effect sizes cannot be pooled, study‐level effects will be reported in as much detail as possible. Software for storing data and statistical analyses will be RevMan 5.0 and EPPI reviewer.

#### 3.4.7 Unit of analysis issues

The unit of analysis of interest to the present study is people with disabilities, their caregivers, or those working with them. Should we encounter a multiarm study, we will pay caution to ensure that the same group of participants is not included twice in analysis. In addition, paired data will be analysed appropriately.

#### 3.4.8 Dealing with missing data

Where the study report is missing key data, the reviewers will attempt to calculate the required measures from reported data (e.g., calculating SE from CIs or *p* value). However, if this is not possible, the author(s) of the original study will be contacted. We will document correspondence with study authors. In the final review, the issue of missing data and their potential impact on the findings will be discussed in the discussion section.

#### 3.4.9 Assessment of heterogeneity

We will examine heterogeneity both in the subject matter of included studies (context, intervention and outcomes) and in the reported effect sizes (visually and using *I* ^2^) (Matthie et al., 2020). If meta‐analysis is appropriate, we will calculate an inverse variance weighted average effect size using a random effects model. However, if there is too much heterogeneity in the reporting of quantitative data, and the effect sizes, we will synthesise the data only narratively, and without a meta‐analysis. Heterogeneity will be assessed by comparing study characteristics such as type of intervention and control comparators, participant demographics, quality of trials (randomisation, blinding, losses to follow‐up) and outcomes measured. Statistical heterogeneity will be assessed visually and by examining the *I* ^2^ statistic, which describes the approximate proportion of variation that is due to heterogeneity rather than sampling error. This will be supplemented by the *χ* ^2^ test, where a *p* < .05 indicates heterogeneity of intervention effects. In addition, we will estimate and present *τ* ^2^, along with its CIs, as an estimate of the magnitude of variation between studies. This will provide an estimate of the amount of between‐study variation. Sensitivity and subgroup analyses will also be used to investigate possible sources of heterogeneity.

The findings will be grouped by suboutcomes, that is: sociocultural, economic, recreation, leisure and sports and access to justice. For each suboutcome, a narrative summary will be prepared for the main themes and findings, including consideration of where there is strong evidence for effect, where there are evidence gaps, and the quality of the evidence. We will conduct a meta‐analysis of results by subgroup if there are sufficient number of studies (*n* = 4, (Fu et al., 2011) and the level of heterogeneity is not too high.

#### 3.4.10 Assessment of reporting biases

Assessment of reporting biases is covered under the section above “assessment of risk of bias in included studies”.

#### 3.4.11 Data synthesis

If there are two or more studies with common characteristics which can be meaningfully and logically grouped together, meta‐analysis will be carried out. EPPI reviewer will be used to synthesise the main effects across all identified studies, and for each outcome area. This will include weighted mean effect size, SE and CI. Forest plots will be used to display findings. In the event that there are not sufficient studies to undertake meta‐analysis, a narrative synthesis will be undertaken and reported.

#### 3.4.12 Subgroup analysis and investigation of heterogeneity

Given that we do not anticipate high‐enough quality quantitative findings to enable a meta‐analysis, we have not planned subgroup analyses as part of a meta‐analysis. However, as noted, we are interested in certain specific populations of people with disabilities, including women, children (particularly vulnerable children, for example, those in care), different impairment groups, conflict (conflict and post‐conflict settings), migrants/refugees/internally displaced people, and ethnic minority groups. For papers addressing these issues, we will extract effect sizes and if data allows, disaggregate outcome findings by group. However, our expectation is that we will instead be able to provide a narrative description of any apparent notable characteristics of papers addressing these groups, but these findings will be descriptive and tentative.

#### 3.4.13 Sensitivity analysis

Treatment of qualitative research

We do not plan to include qualitative research. We will code information on barriers and facilitators from the included studies.

## CONTRIBUTIONS OF AUTHORS

**Content expertise:** Hannah Kuper, Director of the International Centre for Evidence in Disability, a research group at LSHTM that works to expand the research and teaching activities of LSHTM in the field of global disability. Her main research interest is disability in low‐ and middle‐income countries, with a particular focus on assessment of the prevalence of disability and impairments, including in children, and development of new methods in undertaking these surveys (e.g., use of mobile technologies), investigation of the health and rehabilitation needs of people with disabilities, and how these can be met in low resources settings and research on the relationship between poverty and disability, and the potential role of social protection in breaking this cycle. She has an undergraduate degree from Oxford University in Human Sciences and a doctorate from Harvard University in epidemiology. She has worked at LSHTM since 2002.

**Systematic review method and statistical analysis expertise:** All team members have previous experience in systematic review methodology, including search, data collection, statistical analysis, theory‐based synthesis, which mean they are proficient in carrying out the various processes in a systematic review, such as search, eligibility screening, quality assessment and coding. Furthermore, all three authors have experience in statistical analysis of data generated through a systematic review.

**Information retrieval expertise:** All authors have previous experience in developing search strategies.

## DECLARATIONS OF INTEREST

None.

**Preliminary timeframe**

December 2020.

## SOURCES OF SUPPORT

### External sources

- This systematic review is supported by the UK Department of International Development (DFID) under its support for the Centre for Excellence for Development Impact and Learning (CEDIL) and the Programme for Evidence to iNform Disability Action (PENDA), UK.

This systematic review is supported by the UK Department of International Development (DFID) under its support for the Centre for Excellence for Development Impact and Learning (CEDIL) and the Programme for Evidence to iNform Disability Action (PENDA).

## Footnotes

1 Thanks also to Hugh Waddington (3ie and Campbell IDCG) for suggestions used in developing the tool.

2
https://homvee.acf.hhs.gov/HomVEE-Attrition-White_Paper-7-2015.pdf

## APPENDIX A ANNEX 1: CODING TOOL

- Publication status
  o Published
  o Ongoing
- Region
  o East Asia and Pacific
  o Europe and Central Asia
  o Latin America and Caribbean
  o Middle East and North Africa
  o Sub‐Saharan Africa
  o South Asia
- Country (specify)
  o Iran
  o India
  o Albania
  o South Africa
  o Turkey
  o Uganda
  o Indonesia
  o Egypt
  o China
- Type of disability
  o Hearing
  o Physical
  o Visual
  o Intellectual/learning and developmental/behavioural
  o Psychosocial/Mental
  o Can't tell/not reported
- Target group
  o People with disability_Child*Child (0‐17.9 years)*
  o People with disability_adults
  o People with disability_elderly
  o Family member/caregiver
  o Service provider/professional/teachers
  o Community member
  o Other (specify)
- Participants SES
  o Low
  o Medium
  o High
  o Mixed
  o Can't tell/not reported
- Gender of target group
  o Male
  o Female
  o Both
  o Can't tell/not reported
- Study design
  o Randomised controlled trial
  o Controlled before and after
  o RDD
  o ITS
  o Matched designs
  o Others
- Subject assignment
  o Individual random
  o Whole group random
  o Individual matched random
  o Nonmatched and nonrandom
  o Other (specify)
  o Can't tell/not reported
- Geographical setting of the interventions
  o Urban
  o Rural
  o Mixed
  o Can't tell/not reported
- Interventions
  o Personal assistance
    ▪ Formal personal assistance and support (including training): Formal assistance may be provided on a formal basis by governmental and nongovernmental organizations and the private sector. Allowances, such as disability pensions, guardianship awards or caregiver allowances, may be available to fund personal assistance (Khasnabis, Al Jubah, Brodtkorb, Chervin, P. Goerdt).
    ▪ Informal personal assistance and support (including training): *Informal assistance includes assistance by family members, friends, neighbours and/or volunteers (Khasnabis, Al Jubah, Brodtkorb, Chervin, P. Goerdt)*.
  o Relationship, marriage and family
    ▪ Networking and social support: *Includes linking people with disabilities to appropriate support networks in the community, for example, disabled people's organizations and self‐help groups (Khasnabis, Al Jubah, Brodtkorb, Chervin, P Goerdt)*.
    ▪ Improving community attitude: *It involve working with the media to promote positive images and role models of people with disabilities; and information on services available (Khasnabis, Al* Jubah*, Brodtkorb, Chervin, P Goerdt)*.
    ▪ Community living: *It involves interventions to support people with disabilities to access their preferred living arrangements and support people with disabilities who are homeless to find* appropriate *accommodation, preferably in the community*.
    ▪ Social and communication skill training: *Social skills training is a therapeutic approach used to improve interpersonal relations. The therapy focuses on verbal and nonverbal behaviors* common *in social relationships*.
    ▪ Violence prevention interventions: *This includes all the interventions to prevent violence such as raising awareness, establishing links to local stakeholders for support, access to health* care *services, and so forth (Khasnabis, Al Jubah, Brodtkorb, Chervin, P Goerdt_*.
  o Culture and arts
    ▪ Access and participation in cultural programs, arts, drama and theatres: *People with disabilities enjoy access and participation to cultural materials in accessibleformats; to television programmes, films, theatre and other cultural activities, in accessibleformats; to places for cultural performances or services, such as theatres, museums, cinemas, libraries and tourism services, and, as far as possible, to monuments and sites of national cultural importance (Article 30 of the UN CRPD)*.
    ▪ Access and participation in religious activities: *People with disabilities enjoy access and participation in religious and spiritual activities in accessible formats*, for example, *making prayers, songs, chanting, and* sermons *accessible with signed translation, and making religious texts available in large print, audio and Braille; Places of worship are physically accessible and that religious practices are modified to accommodate people with disabilities (Article 30 of the UN CRPD)*.
  o Recreation, leisure and sports
    ▪ Access and participation in sports events: *This includes strategies that encourages people with* disabilities *to have access and provide opportunities to participate in mainstream sporting activities at all levels through inclusive sports event; have an opportunity to organise, develop and participate in disability‐specific sporting and recreational activities through provision of support and links with DPOs for people with disabilities, assisting them to develop strategic, national and international partnerships and have access to adapted sports equipment (Khasnabis, Al Jubah, Brodtkorb, Chervin, P Goerdt)*.
    ▪ Access and participation in recreation and leisure: *This includes strategies that encourages people with disabilities to have access and provide opportunities to participate in mainstream sporting activities to provide opportunities to participate actively or passively in recreation, tourism and leisure (Khasnabis, Al Jubah, Brodtkorb, Chervin, P Goerdt)*.
  o Access to justice
    ▪ Accessibility of legal system and justice: *“Accessibility”, in this publication refers to a feature or quality of any physical or virtual environment, space, facility or service that is capable of accommodating the needs of people with disabilities to understand, get access to or interact with legal system. Accessibility also refers to technical standards that are mandated nationally or internationally for the design and construction of a physical or virtual environment, space, facility and service. Examples include accessible built infrastructure of courts such as ramps, and so forth*.
    ▪ Access to legal system and justice: *Refers to people's ability to access the systems, procedures, information, and locations used in the administration of justice (*Lord & Stein, 2008 *). This includes activities such as legal awareness through DPOs and media, legal aid*.
  o AT and rehabilitation
    ▪ Assistive technology
    ▪ Rehabilitation: *Rehabilitation is a process intended to eliminate or at least minimize*—*restrictions on the activities of people with disabilities, permitting them to become more independent and enjoy the highest possible quality of life (*Bailey & Angell, 2005 *). This will include activities as provision of mobility, hearing, visual devices, and therapies to use these devices*.
    ▪ Medical care: *Provision of medical services to ensure that people with disabilities can access services designed to identify, prevent, minimize and/or correct health conditions and impairments (Khasnabis, Al Jubah, Brodtkorb, Chervin, P Goerdt)*.
  o Policies and programmes
    ▪ International legislations and policies: *These include international legislations and policies through which countries abolish discrimination against persons with disabilities and eliminate barriers towards the full enjoyment of their rights and their inclusion in society (UN Department of Economic and Social Affairs*.
    ▪ Social inclusion policies: *This includes inclusive policies on employment, educational and provision of housing and accommodation to people with disabilities*.
- Outcomes
  o Social
    ▪ Social identity: *Social identity is defined asthat part of a person's self‐concept which derives fromthe knowledge of* his *or hermembership in a social group(or groups) together with the value and emotional significance attached to that membership. Social identity can spur intergroup discrimination and other formsof intergroup conflict (Simon & Trötschel)*.
    ▪ Personal assistance: *People with disabilities have individual support plans in place, have access to training to enable them to manage their personal assistance needs, or that support is available for families who provide personal assistance on an informal basis*.
  o Skills for social inclusion
    ▪ Social and communication skills: *Kratchowill and French (1984) view social skills as learned verbal and nonverbal behaviour performed within a specific social context of an aggressiveness‐shyness continuum, and view adjustment in relation to an individual's social perceptual accuracy (that is, the ability to understand subtle nuances and define critical elements in social environment). Examples include civic, social engagement and interaction and professional social skills*.
    ▪ Communication skills: *It is the act of transferring information. It may be vocally (using voice), written (using printed or digital media such as books, magazines, websites or emails), visually (using logos, maps, charts or graphs) or nonverbally (using body language, gestures and the tone and pitch of voice). This includes availability and use of communication aids and speech and reading devices*.
    ▪ Social behavior: *Social behavior can be defined as all behavior that influences, or is influenced by, other members of the same species. The term thus covers all behavior that tends to bring individuals together as well as all forms of aggressive behavior (*Grant, 1963 *). This includes conduct problems, peer problems, pro‐social behaviours*.
  o Broad based social inclusion and participation measure
    ▪ Social inclusion*Social inclusion is defined as the process of improving the terms of participation in society*, particularly *for people who are disadvantaged, through enhancing opportunities, access to resources, voice and respect for rights (UN, 2010). These will include measures such as people with disabilities spending more time out of the house, and travelling further away from the house (as well as earning more and spending less time begging)*.
    ▪ Community integration: *Community Integration is the opportunity to live in the community and be valued for one's uniqueness and abilities, like everyone else (Salzer*, 2008 *). Community integration is designed to help people with disabilities to optimise their personal, social, and vocational competency to live successfully in the community*.
    ▪ Community participation*People with disabilities have access, accessibility and opportunities to participate in community activities such as leisure activities, such as hobbies, arts, and sports, political and civic activities or organizations and productive activities, like employment or education; consumption, or access to goods and services; religiousand cultural activities and groups (McConkey*, 2007; *Verdonschot et al.*, 2009).
    ▪ Access to justice*People with disabilities get access to or interact with legal system*
  o Relationships
    ▪ Interpersonal and Family relationship*People with disabilities valuerelationships with family members, staff, friends, acquaintances, and intimate partners (*Clarkson et al., 2009 *) and other peoplewith disabilities (McVilly et al.*, 2006b *), and feeling a sense of belonging to a network when they have different people fulfilling* different *needs (McVilly et al.*, 2006b *). This also includes aspects of participation in household, behaviour of the family towards the people with disability (e.g., more sensitive to child's interests, responded moreappropriately, expressed more warmth)*
    ▪ Peer and community relationships*Community members are aware and accept that people with disabilities can have meaningful* relationships*, marry and have children. (Community‐Based Rehabilitation: CBR Guidelines)*
    ▪ Violence and abuse*People with disabilities are protected against violence, and all relevant stakeholders work together to address the issue. (Community‐Based Rehabilitation: CBR Guidelines)*
- Duration of study (specify)
- Duration of interventions (specify?)
- Upon what kind of statistical analysis were the major findings of the study based?’
  o Descriptive analysis
  o t‐test
  o ANOVA
  o ANCOVA
  o Regression
  o Other (specify)
- Intervention was delivered by?
  o Intervention therapist/coach/occupational therapist
  o Community members
- Sample size of intervention group
- Sample size of control group
- Outcomes details
  o Are descriptive statistics reported for the primary outcome?
    ▪ Yes
      ▪ If yes, please add for the intervention* groupDescriptive statistics for the intervention group. *If there is more than one intervention group please add this below.
      ▪ Number (n)What is the number for the intervention group in the data analysed for this outcome? Add numeric data only to the info box.
      ▪ Pretest meanPlease record the pretest mean (if provided) for the intervention group for this outcome. Add numeric data only to the info box.
      ▪ Pretest standard deviationPlease record the pretest standard deviation (if provided) for the intervention group for this outcome. Add numeric data only to the info box.
      ▪ Posttest meanPlease report the posttest mean for this outcome for the intervention group (if provided) for this outcome. Add numeric data only to the info box.
      ▪ Post test standard deviationPlease record the posttest standard deviation for the intervention group for this outcome (if provided). Add numeric data only to the info box.
      ▪ Gain score mean (if reported)Please add the gain score (pre‐ to posttest) mean for the intervention group. Add numeric data only to the info box.
      ▪ Gain score standard deviation (if reported)Please add the gain score (pre‐ to posttest) standard deviation for the intervention group. Add numeric data only to the info box.
      ▪ Any other information?Please add any other statistical information reported about this outcome for the intervention group (e.g., SE), or use to add notes about the numeric data in the categories above.
    ▪ If yes please add for the control group*Descriptive statistics for the intervention group*
      ▪ Number (n)What is the number for the control group in the data analysed for this outcome? Add numeric data only to the info box.
      ▪ Pretest meanPlease record the pretest mean (if provided) for the control group for this outcome. Add numeric data only to the info box.
      ▪ Pretest standard deviationPlease record the pretest standard deviation (if provided) for the control group for this outcome. Add numeric data only to the info box.
      ▪ Posttest meanPlease report the posttest mean for this outcome for the control group (if provided) for this outcome.
      ▪ Post test standard deviationPlease record the posttest standard deviation for the control group for this outcome (if provided).
      ▪ Gain score mean (if reported)Add numeric data only to the info box.
      ▪ Gain score standard deviation (if reported)Add numeric data only to the info box.
      ▪ Any other information?Please add any other statistical information reported about this outcome for the intervention group (e.g., SE).
    ▪ No
  o Is there follow up data?*Please provide details of any assessment to measure long lasting effects (e.g., delayed posttest or long term follow up)*
    ▪ Yes
    ▪ No
- Critical appraisal
  o Study design (Potential confounders taken into account)
    ▪ LOW: Before versus after. Naïve matching
    ▪ MEDIUM: IV, RDD, PSM, double difference
    ▪ HIGH: RCT, natural experiment
  o Blinding (RCTs only)
    ▪ LOW: No mention of blinding
    ▪ MEDIUM: Blinding for analysis
    ▪ HIGH: Blinding of data collection (where feasible) and blinding for analysis
  o Losses to follow up are presented and acceptable
    ▪ LOW: Attrition not reported, OR falls well outside WWC acceptable combined levels*
    ▪ MEDIUM: Overall and differential attrition close to WWC combined levels*
    ▪ HIGH: Overall and differential attrition within WWC combined levels*
  o Disability/impairment measure is clearly defined and reliable
    ▪ LOW: No definition OR overall attrition > 50%
    ▪ MEDIUM: Unclear definition OR Single question item only (e.g., are you disabled)
    ▪ HIGH: Clear definition, for example, Washington Group questions, detailed measure of impairment
  o Outcome measures are clearly defined and reliable
    ▪ LOW: No definition
    ▪ MEDIUM: Unclear definition
    ▪ HIGH: Clear definition using existing measure where possible
  o Baseline balance (N.A. for before vs. after)
    ▪ LOW: No baseline balance test (except RCT) OR reported and significant differences on more than five measures. PSM without establishing common support.
    ▪ MEDIUM: Baseline balance test, imbalance on 5 or fewer measures
    ▪ HIGH: RCT, RDD
  o Overall confidence in study findings
    ▪ LOW: Low on any item
    ▪ MEDIUM: Medium or high confidence on all items
    ▪ HIGH: RCT with high confidence on all items
